# Leadership and international collaboration on COVID-19 research: reducing the North–South divide?

**DOI:** 10.1007/s11192-023-04754-x

**Published:** 2023-06-05

**Authors:** Danilo Silva Carvalho, Lucas Lopes Felipe, Priscila Costa Albuquerque, Fabio Zicker, Bruna de Paula Fonseca

**Affiliations:** 1 Center for Technological Development in Health (CDTS), Oswaldo Cruz Foundation (Fiocruz), Av. Brasil 4036, Rio de Janeiro, RJ 21040-361 Brazil; 2 Post Graduation Program in Informatics (PPGI), Department of Computer Science, Federal University of Rio de Janeiro (UFRJ), Cidade Universitária, Av. Athos da Silveira Ramos 274, Rio de Janeiro, RJ 21941-916 Brazil

**Keywords:** COVID-19, Research collaboration, Networks, Leadership, Low- and middle-income countries, Bibliometrics

## Abstract

The COVID-19 pandemic triggered unprecedented scientific efforts worldwide and launched several initiatives to promote international cooperation. Because international scientific collaborations between high-income countries (HICs) and low- and middle-income countries (LMICs) are not always balanced, analyzing research leadership helps to understand the global dynamics of knowledge production during COVID-19. In this study, we focused on HIC–LMIC collaborations on COVID-19 research in 469,937 scientific publications during the first 2 years of the pandemic (2020–2021). Co-authorship and authors’ affiliation were used to identify international collaborations, according to country income level. The leadership analysis considered the countries of the first and last authors of publications. The results show that (i) most publications with international collaborations (49.3%) involved researchers from HICs and LMICs; (ii) collaborative research between HICs and LMICs addressed relevant public health needs; (iii) HIC–LMIC collaborations were primarily led by researchers from the United States, China, the United Kingdom, and India; (iv) most HIC–LMIC publications (44%) had shared leadership, with research interests linked to national expertise and global interests. This study contributes to the analysis of research collaborations on COVID-19 and sheds light on North–South relations in the production and dissemination of scientific knowledge.

## Introduction

Scientific research collaborations follow the evolution, development, and the multidisciplinarity of science: one in five published articles is the result of an international collaboration (NSF, 2019). International collaborations allow scientists to acquire complementary expertise that transcends national boundaries and internationalize their research, ultimately leading to greater impact (Wagner & Jonkers, 2017).

International health challenges such as the COVID-19 pandemic often draw national efforts (Bump et al., 2021). Several initiatives to promote international scientific collaboration have emerged during this period (Budd et al., 2020). Research findings were made widely and freely available, comprehensive datasets were created and shared, and there were specific calls for collaboration. The scientific community responded quickly by sharing new ideas, data, and results (Maher & Van Noorden, 2021).

Participation in scientific collaboration networks enables the development and strengthening of national research capacity in low- and middle-income countries (LMICs), which is critical for addressing health challenges (Thorsteinsdóttir et al., 2011). COVID-19 posed particular challenges for LMICs because their health systems are often underfunded and they have limited influence on the global health research agenda (Norton et al., 2021). Although some LMICs have played a key role in generating and disseminating new knowledge about epidemics (Machado-Silva et al., 2019; Vasconcellos et al., 2018), recent studies have shown that their participation in COVID-19 international collaborations was low (Fry et al., 2020) and that they made a small contribution to the advancement of knowledge in this area (Pamplona da Costa et al., 2021).

Scientific leadership in research can be understood as the responsibility for conducting and/or coordinating research development (González-Alcaide et al., 2017). In biomedical and health science publications, the order of authors usually expresses research leadership, often positioned as the first and last author (Abramo et al., 2013; Hedt-Gauthier et al., 2019). Economics and power asymmetries may influence authorship and leadership relationships, affecting the representation of LMIC researchers in scientific publications (Hedt-Gauthier et al., 2019). A previous analysis of leadership in health research found that LMIC researchers are less likely to hold key authorship positions in international collaborations (González-Alcaide et al., 2017), particularly when their coauthors are from high-income countries (HICs) (Hedt-Gauthier et al., 2019). A recent study showed that one-fifth of COVID-19 publications relevant to Africa had no African authors and 66.1% of authors of African publications were not from Africa (Naidoo et al., 2021). Global links to research production remain unchanged despite substantial growth in health research in LMICs (Franzen et al., 2017).

In this study, we examine international research collaboration on COVID-19 during the first 2 years of the pandemic (2020–2021), focusing on HIC–LMIC collaboration. Our goal was to assess the scope of HIC–LMIC research to public health, map patterns of leadership, and identify the flow of scientific knowledge between partner countries across income groups. The evolution of leadership over time and the major research themes for different types of leadership were also examined. This study contributes to the ongoing interest in COVID-19 research collaborations (Cai et al., 2021; Fry et al., 2020) and to understand the North–South balance in the production and dissemination of scientific knowledge.

## Method

### Data selection, extraction, and processing

COVID-19 scientific articles and preprints published between January 1, 2020, and December 31, 2021, were retrieved from the Dimensions database (Digital Science & Research Solutions Inc.) using the Google BigQuery (GBQ) interface^1^ in standard SQL language.^2^ The original database containing articles, preprints, clinical trials, research grants, and research records related to COVID-19 has been previously used for “real-time bibliometrics” (Hook et al., 2021). A dataset containing the selected publications was created on GBQ on April 1, 2022. Only records that included the DOI, institutional affiliation of authors, and country were included. The indexing date in the Dimensions database was used as a reference for normalizing the date between online and print publications. National publication contributions were calculated using the full count method. Monthly growth rate estimates of COVID-19 publications were based on publications indexed in January 2020.

### Characterization of international collaboration

Co-authorship of scientific publications was considered a proxy for scientific collaboration. The country of institutional affiliation of all authors, classified by the World Bank as LMICs and HICs (World Bank, 2020), was used to identify international collaborations. Overseas territories and islands were assigned to the corresponding countries. Authors belonging to institutions in England, Scotland, Northern Ireland, and Wales were collectively referred as the United Kingdom.

Three groups of international collaborations were considered: (i) HIC–HIC (when all authors were affiliated with HIC institutions); (ii) LMIC–LMIC (when all authors were affiliated with LMIC institutions); (iii) HIC–LMIC (when at least one author was affiliated with a HIC institution and at least one other author was affiliated with an LMIC institution).

### Identifying research leadership

In the leadership analysis, only publications jointly authored by countries in different income groups (HIC–LMIC publications) were considered. The countries of the first and last authors were used as indicators of scientific leaders. Therefore two leading scientists and their respective countries of affiliation were profiled in each publication. Leadership roles were classified as: (i) HIC leadership—when both the first and last authors were affiliated with HIC institutions, (ii) LMIC leadership—when both the first and last authors were affiliated with LMIC institutions, and (iii) Shared leadership—when the first and last authors were alternately affiliated with either an LMIC or HIC institution.

### Assessing knowledge flow and scientific influence

Knowledge flow and influence were assessed exclusively in HIC–LMIC collaborative publications, regardless of the leading country. The scientific influence was assessed from a social network analysis (SNA) perspective, as leading countries have a greater influence on collaborations (González-Alcaide et al., 2017). The influence was measured by estimating the outdegree centrality, which is an indicator of knowledge outflow from one country to another. Outdegree centrality measures the number of outgoing links from a given node in a network (Wasserman & Faust, 1994). A high outdegree centrality indicates that knowledge frequently flows out from a particular leading country.

Co-authorship relationships were mapped for each publication considering direct links between the first and last author’s countries to all other countries. A list of lead-partners was imported into the Gephi software (Bastian et al., 2009) to create and visualize network graphs and calculate metrics. In these directed networks, nodes represent countries, and two or more countries were connected if their associated researchers had a lead-collaboration relationship in one or more publications. For visualization, the size of a node was proportional to its outdegree centrality, and the thickness of the links between them indicated the strength of the collaboration. Green nodes represent HICs and orange nodes represent LMICs. For spatial visualization of the network, the country affiliation data were manually geocoded and processed using the “GeoLayout” and “Map of Countries” plugins available in Gephi.

### Thematic mapping and clustering

Term maps were created using the VOSviewer 1.6.18 software (Centre for Science and Technology Studies, Leiden University, the Netherlands). Terms were extracted from the titles and abstracts of all HIC–LMIC collaborative publications and clustered according to their similarity using the “association strength” measure proposed by Van Eck and Waltman (Van Eck & Waltman, 2009). Only terms that appeared at least 50 times in the titles and abstracts were considered. The terms “SARS-Cov2” and “coronavirus” were excluded to improve cluster visualization. The higher the number of publications in which two terms appeared together, the stronger their association, and the closer they were placed on the map. Graphically, each term was represented by a circle whose diameter and label size were proportional to the number of occurrences of the corresponding term in the title or abstract of publications.

Clusters of terms were classified by two independent researchers according to WHO’s Health Topics (WHO, n.d.) (Table 1). Discordant classifications were reviewed by a third researcher, and a final classification was agreed upon by consensus.

**Table 1:** COVID-19 research topic clusters and associated terms

Cluster	Topics	Associated terms (e.g.)
1	Clinical Medicine	Mortality, severity, pneumonia, complication, diabetes
2	Mental Health	Anxiety, depression, stress, perception, attitude
3	Economy, Investments, and Sustainability	Crisis, production, industry, market, economy
4	Education	Student, education, organization, opportunity, university
5	Diagnostic Assays	Detection, variant, antibody, sensitivity, mutation
6	Biomedical Engineering	Application, property, surface, sensor, efficiency
7	Immunopathology	Cell, therapy, pathway, gene, expression
8	Transmission Dynamics	Concentration, temperature, emission, air quality, aerosol
9	AI, Technology, and IoT	Technique, feature, framework, algorithm, dataset
10	Drug R&D and Repurposing	Drug, interaction, protein, structure, compound
11	Clinical Trials	Support, consultancy, trial, efficacy, safety

## Results

### International collaboration has increased but with a limited share of publications

**Fig. 1:**
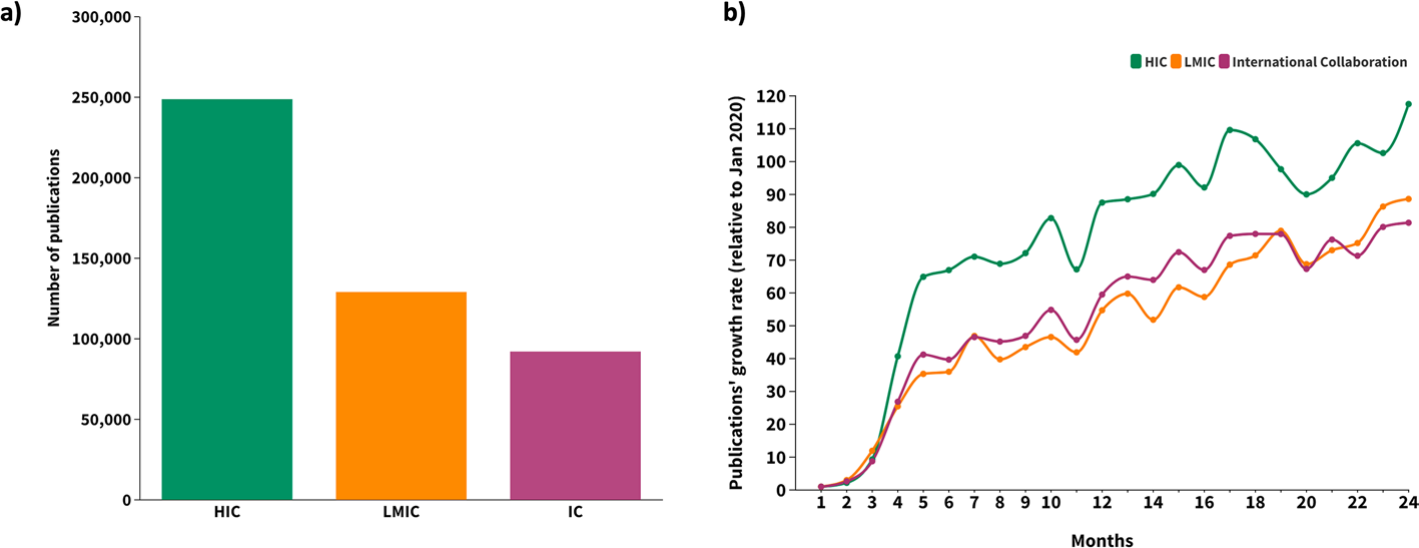
Scientific publications on COVID-19 and international collaboration by country income level (2020–2021). **a** Total number of publications. HIC: all authors were affiliated with institutions in a single HIC; LMIC: all authors were affiliated with institutions in a single LMIC; IC: International collaboration: authors were affiliated with institutions based on at least two different countries, whether HIC or LMIC; **b** Publication growth rate (%). *HIC* high-income country, *LMIC* low- and middle-income country, *IC* international collaboration

A total of 469,937 publications, 432,814 articles (92.2%), and 37,123 preprints (7.8%) were analyzed. Most publications were produced by a single HIC (*n* = 248,596—52.9%) or LMIC (*n* = 129,232–27.5%), without international collaboration (Fig. 1a). Publications in international collaboration doubled between the first and second year (Fig. 1b) and accounted for 19.6% (*n* = 92,085) of the total articles analyzed. Compared to the first month of the pandemic, a 50-fold growth rate of publications was observed in 6 months, mainly on account of HICs (Fig. 1b). The overall participation of LMICs in COVID-19 international collaborative research has increased steadily during this period, albeit at a lower rate than HIC publications (Fig. 1b).

### Collaboration between HICs and LMICs accounted for nearly half of all international collaborations

**Fig. 2:**
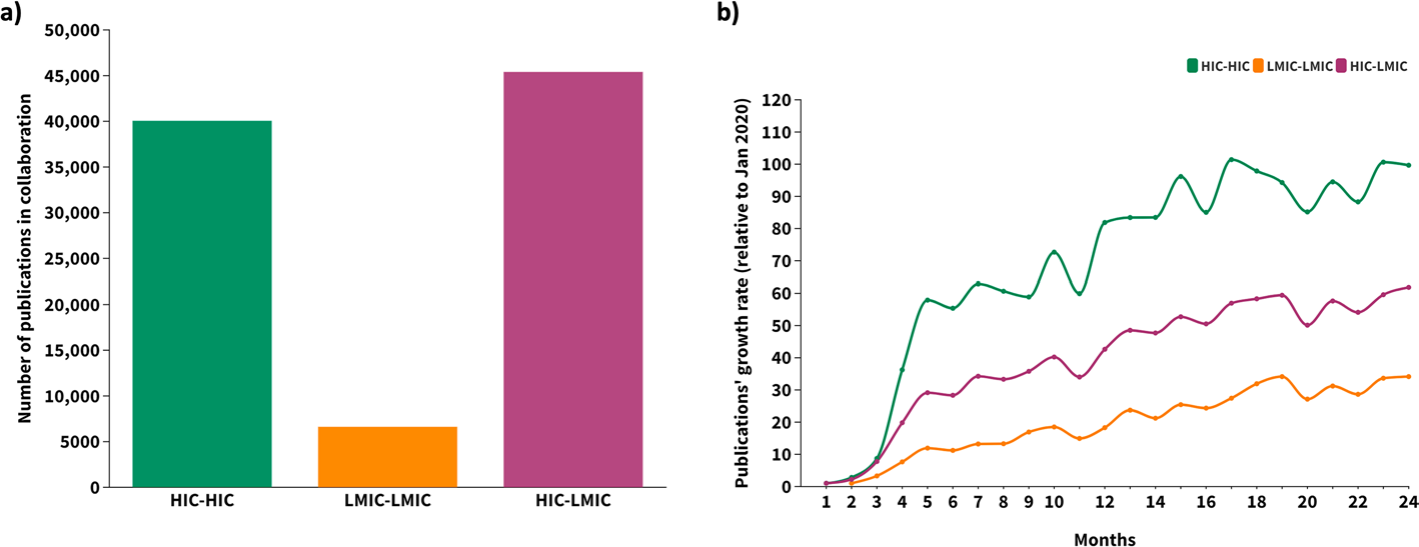
International collaborative publications on COVID-19 (2020–2021). **a** Total number of publications. HIC–HIC: all authors were affiliated with HIC institutions; LMIC–LMIC: all authors were affiliated with LMIC institutions; (iii) HIC–LMIC: at least one author was affiliated with a HIC institution and at least one other author was affiliated with an LMIC institution; **b** Publication growth rate (%). *HIC* high-income country, *LMIC* low- and middle-income country

Almost half of all international collaborations (49.3%, *n* = 45,397) were published jointly by HIC–LMIC authors (Fig. 2a). HIC–HIC collaborations accounted for 43.5% (*n* = 40,056) of publications and LMIC–LMIC collaborations accounted for only 7.2% (*n* = 6630) (Fig. 2a). In the 2 years considered, HIC–LMIC and LMIC–LMIC publications grew at similar rates, with a slight increase in HIC–LMIC publications.

Overall, the United States of America (USA) and China were the most frequent collaborators, accounting for 12.6% (*n* = 11,658) of the total international collaborative publications, followed by the USA and Canada (11.7%) and the USA and the United Kingdom (7.8%) (Table 2). The most frequent HIC–HIC collaborations involving the USA were with the United Kingdom (23.9%), Canada (18.6%), and Italy (10.3%). LMIC–LMIC co-authorship was more frequent in Asia, particularly between China and Pakistan (15.3%), China and Taiwan (9.4%), and India and Thailand (8.5%). HIC–LMIC collaboration was more frequent between the USA and China (25.6%), followed by USA and India (11.4%), the United Kingdom and China (10.6%), and USA and Brazil (8.1%). Notably, the USA and the United Kingdom frequently co-occur in HIC–LMIC publications and were the fourth most frequent partners in this dataset (Table 2).

**Table 2:** Top international collaboration partners in COVID-19 publications

Collaboration type	Rank	Collaborating countries	Number of publications (%)
All	1	USA–China	11,658 (12.6)
	2	USA–Canada	10,080 (11.7)
	3	USA–United Kingdom	7,201 (7.8)
	4	USA–Australia	6,466 (7.0)
	5	USA–Italy	6,104 (6.6)
HIC–HIC	1	USA–United Kingdom	9,602 (23.9)
	2	USA–Canada	7,440 (18.6)
	3	USA–Italy	4,126 (10.3)
	4	USA–Germany	4,102 (10.2)
	5	USA–Australia	3,956 (9.9)
LMIC–LMIC	1	China–Pakistan	1,014 (15.3)
	2	China–Taiwan	624 (9.4)
	3	India–Thailand	566 (8.5)
	4	China–India	526 (7.9)
	5	Malaysia–Indonesia	374 (5.6)
HIC–LMIC	1	USA–China	11,658 (25.6)
	2	USA–India	5,192 (11.4)
	3	United Kingdom–China	4,840 (10.6)
	4	USA–United Kingdom	4,800 (10.5)
	5	USA–Brazil	3,662 (8.1)

### Research topics change according to the type of collaboration

**Fig. 3:**
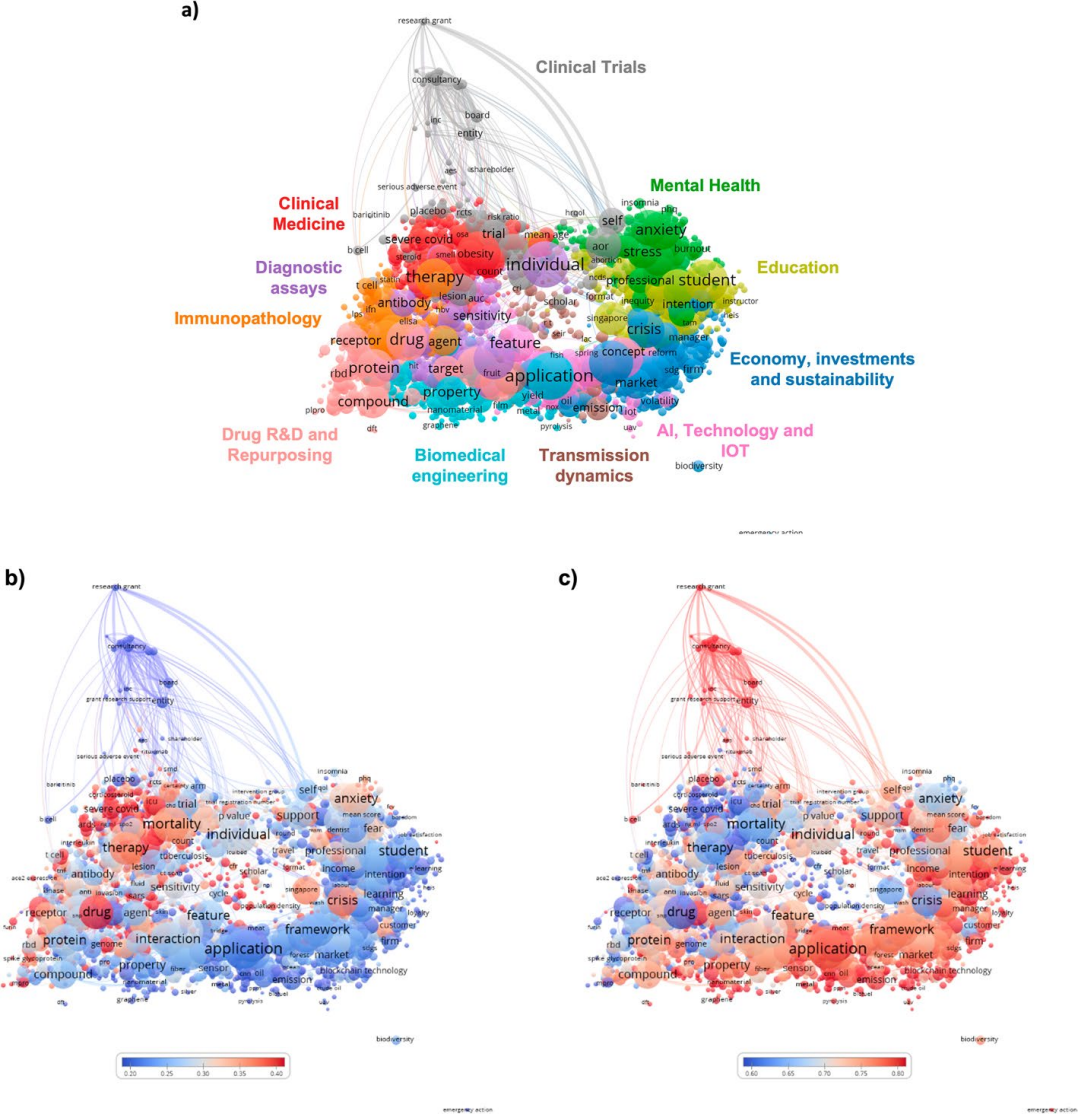
COVID-19 research themes in HIC–LMIC publications. Terms were extracted from titles and abstracts of HIC and LMIC co-authored publications. The closer two terms are positioned to each other, the stronger their relationship. Terms were represented by circles, with the diameter and label size proportional to their frequency in the dataset. **a** Different colors distinguish clusters of terms that have co-occurred more frequently. **b**, **c** Colors indicate the degree of occurrence of a term during the first (**b**) and second (**c**) year of the pandemic, relative to the overall period. Blue represents a low occurrence and red a high occurrence. (Color figure online)

The term map shows 2,688 terms extracted from the titles and abstracts of 45,115 HIC–LMIC publications (99.3% of all HIC–LMIC publications). The map shows 11 major research topics (Fig. 3a): Clinical Medicine (red); Clinical trials (gray); Mental Health (green); Education (yellow); Economy, investments, and sustainability (blue); Artificial intelligence, technology and internet of things (IoT) (pink); Transmission dynamics (brown); Biomedical engineering (turquoise); Drug R&D and Repurposing (salmon); Immunopathology (orange); and Diagnostic assays (purple).

The dynamics of research interests during the study period are illustrated by an overlay of the most common terms in each year. Terms were colored according to their score for each year relative to the entire 2-year period, from blue (lowest score) to red (highest score) (Fig. 3b, c). In the first year of the pandemic (2020), clinical, mental health, and drug discovery research studies had the highest scores (Fig. 3b). In the second year (2021), clinical trials, and research in education, economics, artificial intelligence, transmission dynamics, and biomedical engineering were the most common (Fig. 3c).

### The USA, China, the United Kingdom, and India have had the greatest influence on COVID-19 research in HIC–LMIC collaborations

**Fig. 4:**
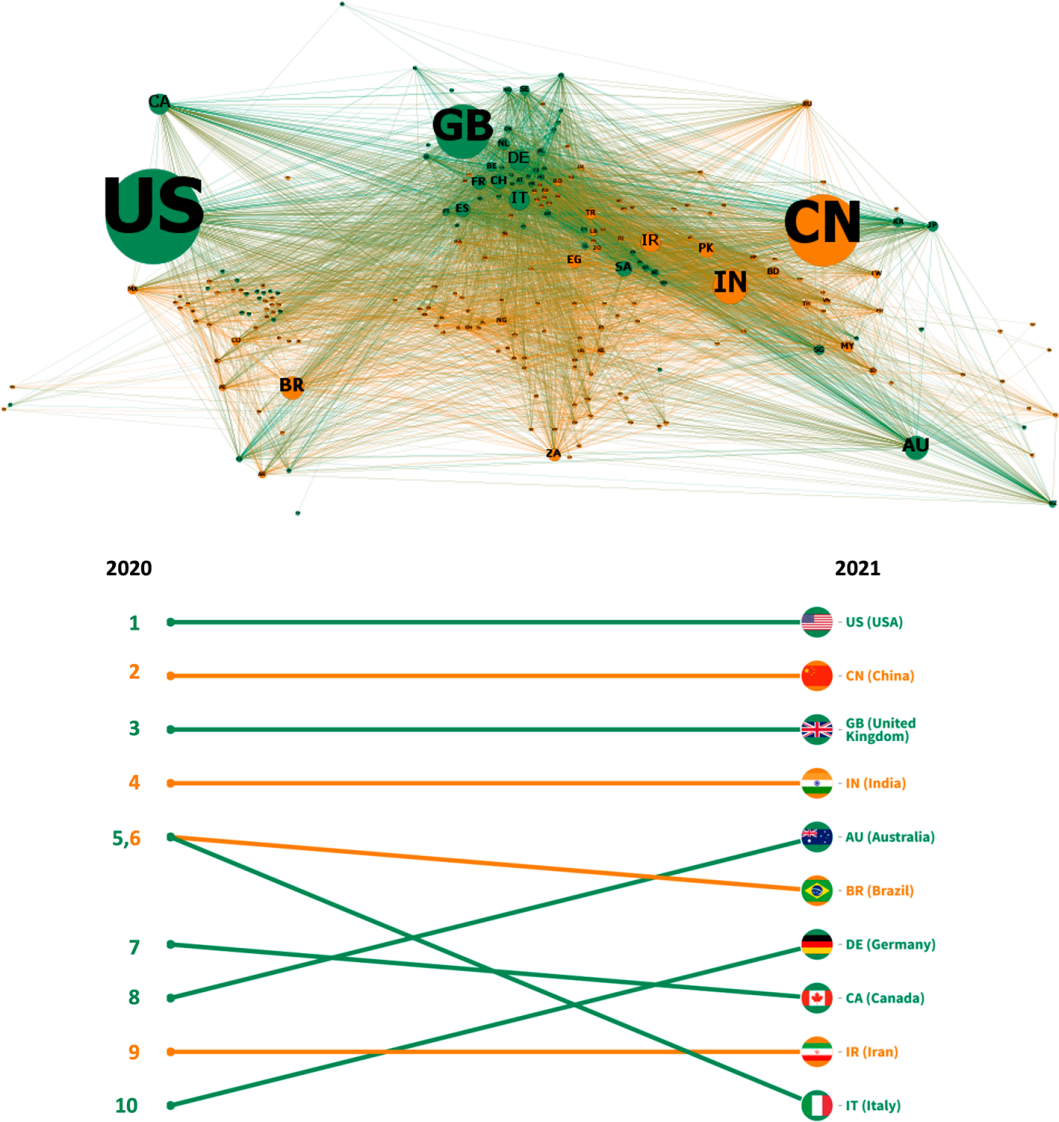
Most influential countries in HIC–LMIC COVID-19 publications. Upper panel: HIC–LMIC collaboration network. Each node represents a country, linked to other countries by the co-authorship of publications. Nodes are color-coded as orange (LMIC) or green (HIC). The size of the node is proportional to its outdegree centrality. Lower panel: Ranking of countries by outdegree centrality over the study period. (Color figure online)

The metric of outdegree centrality was used as a proxy for country influence (knowledge outflow) in HIC–LMIC collaborations. The top panel of Fig. 4 shows the HIC–LMIC research collaboration network. The most influential countries are represented by larger nodes. The USA, China, India, and the United Kingdom (GB) were the countries with the highest outdegree centrality. Trends in the outdegree centrality rankings (bottom panel) show that these four countries maintained their influence in both years.

### Most HIC–LMIC collaborations were the result of shared leadership between countries

**Fig. 5:**
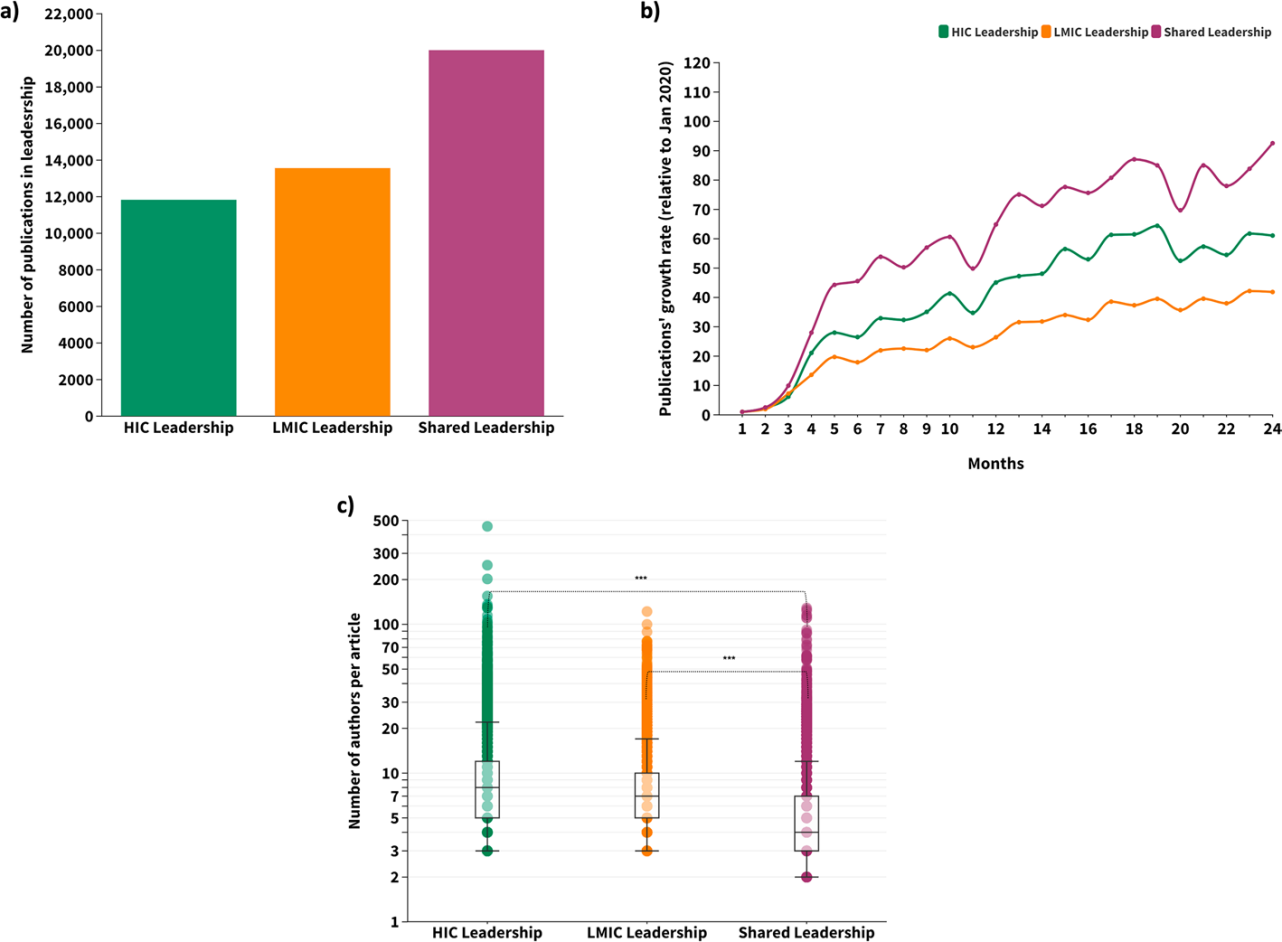
Leadership in COVID-19 publications co-authored by HIC and LMIC researchers (2020–2021). **a** Total number of publications. **b** Monthly growth rate (%) relative to publications indexed in January 2020. **c** Median number of authors per publication. HIC leadership: first and last authors are affiliated with HIC institutions; LMIC leadership: first and last authors are affiliated with LMIC institutions; Shared leadership: the first and last authors are distinctly affiliated with institutions from HIC and LMIC

From the HIC–LMIC publications (*n* = 45,397), most were the result of shared leadership between countries, with the first and last authors distinctly affiliated with HIC or LMIC institutions (*n* = 19,974–44%) (Fig. 5a). The growth rate of publications under shared leadership increased more than a thousand-fold over the study period (Fig. 5b). HIC–LMIC collaborative publications were led exclusively by HICs in 26.0% (*n* = 11,806) and by LMICs in 29.9% (*n* = 13,576) (Fig. 5a).

From the publications with shared leadership (*n* = 20,011), 64% (*n* = 12,820) of the first authors were affiliated with an LMIC institution and 35.9% (*n* = 7191) with a HIC institution (data not shown). The number of co-authors in publications varied by type of leadership: HIC–LMIC publications under shared leadership had significantly fewer authors (median = 4, mean = 5.7) than publications led solely by HICs (median = 8 and mean = 10.4) or LMIC authors (median = 7, mean = 8.6) (Fig. 5c).

Researchers from the USA and China were the most frequent leading partners in publications with shared leadership (8.3%, *n* = 1655). Other frequent partners were India and USA (*n* = 767, 3.8%), China and the United Kingdom (*n* = 636, 3.2%) (Table 3).

**Table 3:** Leading country partners in COVID-19 shared leadership publications

Rank	Leading countries	Publications (%)
1	China & USA	1,655 (8.3)
2	India & USA	767 (3.8)
3	China & United Kingdom	636 (3.2)
4	China & Australia	418 (2.1)
5	India & United Kingdom	364 (1.8)
6	Brazil & USA	350 (1.7)
7	China & Canada	305 (1.5)
8	Iran & USA	298 (1.4)
9	India & South Africa	280 (1.3)
10	Pakistan & South Africa	240 (1.2)

### Research interests were influenced by the leading authors’ profile

**Fig. 6:**
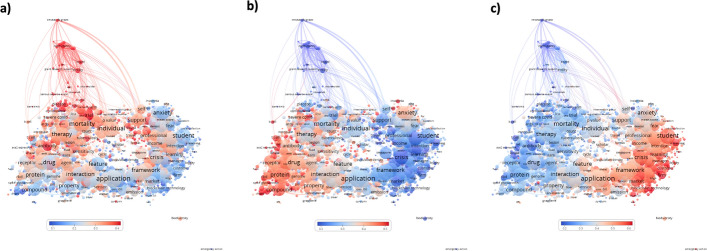
Dynamics of COVID-19 research themes according to the type of leadership in HIC–LMIC publications. Terms were extracted from titles and abstracts of publications co-authored by HIC and LMIC. The closer two terms are located to each other, the stronger their relationship. Terms are represented by circles, with the diameter and label size proportional to their frequency in titles or abstracts. Colors indicate the degree of occurrence of a term in HIC-led publications (**a**), LMIC-led publications (**b**), or in shared leadership (**c**)

Research interests varied significantly by the leadership profile (Fig. 6). Most publications led by HIC focused on clinical trials, clinical medicine, and diagnostic assays (Fig. 6a), while LMIC-led publications focused on drug discovery and drug repurposing, as well as select mental health topics. Immunopathology was a common research interest (Fig. 6b). Publications under shared leadership addressed different topics, including economics, investments and sustainability, education, artificial intelligence, technology and the Internet of things, and mental health (Fig. 6c).

## Discussion

In response to WHO’s declaration of a public health emergency of international concern (PHEIC), researchers tend to respond quickly with a substantial increase in scientific publications, especially when their countries/regions are affected (Zhang et al., 2020). The results presented here confirm this trend with COVID-19.

In the first 2 years of the COVID-19 pandemic, international collaborations accounted for 19.6% of publications. In the early years of an epidemic, international collaborations tend to be low, as seen with Severe Acute Respiratory Syndrome (SARS) in 2003, Influenza A (H1N1) in 2009, Middle East Respiratory Syndrome (MERS) in 2012, and the Ebola outbreak in 2014 (Wu et al., 2021). Collaboration increased over time, reaching 25% for H1N1, 30% for SARS, up to 42% for MERS, and 50% for Ebola (Wu et al., 2021). When scientifically advanced countries respond to non-local public health emergencies, international collaboration with less scientifically advanced countries is to be expected (Wu et al., 2021). A previous study showed that early research on COVID-19 involved less international collaboration and smaller teams than pre-pandemic coronavirus research (Fry et al., 2020). Despite calls for more collaboration, researchers seemed to prefer smaller, multidisciplinary teams to avoid the coordination costs associated with international research (Cunningham et al., 2021).

Our results show that HIC–LMIC collaborations accounted for a large proportion of international collaborations, mostly influenced by the high share of joint articles between the USA and China. Other HIC–LMIC collaborations facilitated the initiation and execution of COVID-19 research in LMICs, including better access to funding, knowledge, and experimental treatments (Bassi et al., 2020). Collaborations between HIC–LMIC have also supported studies on the impact of sociocultural beliefs that have affected the sustainability of social distancing or lockdown in some countries, as well as greater dissemination of research findings in LMICs’ local newspapers and open-access journals (Fanning et al., 2021).

Recently, several studies have been published on COVID-19 research collaboration (Cai et al., 2021; Fry et al., 2020). Previous research has shown that few LMICs were involved in early COVID-19 research, but when these countries were affected by the pandemic, there were an increasing volume of publications (Cai et al., 2021; Fry et al., 2020). Analysis of international collaboration networks revealed an initially sparse network that evolved into a more structured and dense network, similar to the period before COVID-19 (Cai et al., 2021). Although these studies have discussed international collaboration in COVID-19 research, they have not examined the scientific contributions of these efforts, nor have they addressed authorship or leadership in these publications. Because international scientific collaboration between HICs and LMICs is not always a balanced process, analysis of leadership is critical to understanding the global dynamics of knowledge production.

The research topics addressed by HIC–LMIC collaborations provided a scientific view of the pandemic and revealed how research responded to the COVID-19 challenge. Our findings suggest an important contribution to public health. The high level of interest in clinical research in 2020 may be due to initial unawareness of COVID-19 clinical presentation, severity of cases, high-risk comorbidities, and more. Given the urgency posed by the pandemic, repurposing existing drugs appeared to be the most feasible approach to finding treatments for COVID-19 (Guy et al., 2020), as has been reported for antiviral (Khambholja & Asudani, 2020) and anticancer drugs (El Bairi et al., 2020). With the number of virus variants increasing and the pandemic showing no signs of decline, 2021 was marked by international concern about education and the economic impact on society. Issues related to medical education, treatment, prevention, and occupational health and safety (Hou et al., 2021), educational disruption in school and learning (Seetal et al., 2021), effectiveness and attitudes toward online education for children (Ma et al., 2021) and college students (Pelikan et al., 2021) emerged in HIC–LMIC publications. These collaborations also discussed socioeconomic disparities affecting access to health services (Khanijahani et al., 2021) and the economic impact of restrictive policies (Lasaulce et al., 2021). The willingness of LMICs to participate in vaccine and safety, immunogenicity, and efficacy studies (Kitonsa et al., 2021; Pu et al., 2021) is well documented in joint HIC–LMIC publications. Discussions on the ethical aspects of conducting placebo-controlled trials in LMICs (Torres et al., 2021a, 2021b) and the need to strengthen clinical testing capability and infrastructure of LMICs to regulate, manufacture, and distribute medical products (Yamey et al., 2021) have been the subject of HIC–LMIC collaborations with significant impact in the global scientific community.

The major influence of the USA and China in HIC–LMIC research collaborations is not surprising. Since the onset of the pandemic, both countries, which were severely affected, have made important scientific contributions (Fry et al., 2020). Previous studies have also shown that these two countries were central to the global coronavirus research network (Fry et al., 2020; Thavorn et al., 2021). Earlier COVID-19 research showed that collaboration between China and the USA was very high. British researchers joined the international teams in the first few weeks (Fry et al., 2020). Despite the intensification of geopolitical tensions, the collaboration between the two countries continued to increase (Lee & Haupt, 2021).

Although authorship alone does not ensure inclusive and fair collaborations, it is an important indicator of who benefits from research efforts. There are two outstanding findings in joint HIC–LMIC COVID-19 publications: (i) the proportion of publications led exclusively by LMICs was slightly higher than those led exclusively by HICs, and (ii) most publications were the result of shared leadership between HICs and LMICs.

International funding programs have encouraged LMICs to lead projects because of the positive impact this can have on their overall health research systems (GECO, 2020). The high rate of publications under shared leadership suggests that authorship relationships were more balanced, possibly because research motivations and issues on COVID-19 transcended national interests. The pandemic highlighted existing inequalities between countries and set the stage for further inequity (Abimbola et al., 2021). However, given the increase in research productivity during public health emergencies (Zhang et al., 2020) and the dramatic increase in open-access publications during the pandemic (Lee & Haupt, 2021), our findings suggest that the research community overcame power asymmetries and promoted scientific globalism to respond to one of the world’s most complex global health challenges. Because scientific globalism aims to promote knowledge and open science, it is usually greater during a global crisis (Lee & Haupt, 2021; Sá & Sabzalieva, 2018).

Previous research has described a significantly lower number of authors for COVID-19 publications compared with previous coronavirus publications (Cunningham et al., 2021; Fry et al., 2020). The lower number of authors in publications under shared leadership compared with publications under HIC or LMIC leadership suggests that the transaction costs required to coordinate efforts in such international collaborations (agreements on proposals, protocols, work plans, methods, funding, personnel, equipment, and data preparation for publications, etc.) (Landry & Amara, 1998) may be even higher when leaders are from countries of different income groups. Limiting the number of authors participating in research can greatly accelerate the process of writing manuscripts for publication, which ultimately leads to a faster submission and timely dissemination of research results.

Authorship in COVID-19 publications seemed to be more balanced between HICs and LMICs, but research interests varied by type of leadership. Clinical trial publications were largely dominated by HIC authors, possibly related to the high costs and infrastructure required for such trials (*COVID-19—ClinicalTrials.Gov*, n.d.). HIC-led publications also have a strong focus on clinical medicine, consistent with the early spread of the pandemic in HICs and the interest in describing clinical manifestations. LMIC’s interest in evaluating the therapeutic potential of existing drugs is consistent with the leading role of China and India in drug repurposing research (Albuquerque et al., 2022). Publications under shared leadership have largely focused on global issues triggered by the pandemic, with implications for the economy, education, and mental health for HICs and LMICs (Kumar et al., 2021).

A more equitable engagement of HIC and LMIC authors is desirable and would benefit global health at large by (i) addressing global health challenges: many of the most pressing public health problems in the world (infectious diseases, malnutrition, maternal and child mortality, etc.) disproportionately affect LMIC. Advancing research on these issues can lead to the development of more effective tools, strategies and policies to reduce their burden, and improve health outcomes worldwide; (ii) diversifying research perspectives: LMIC researchers can offer unique insights and perspectives that are often overlooked in research conducted primarily by HIC scientists; (iii) increasing research capacity: fair cooperation and partnership can help establish or increase the scientific capacity of LMICs, and ultimately create a more sustainable global research ecosystem.

## Conclusions

This study provides a more detailed look at international scientific collaboration related to the COVID-19 epidemic. We show that in the first 2 years of the pandemic: (i) HIC–LMIC collaborations enabled a rapid scientific response to the public health emergency, despite geopolitical tensions; (ii) collaborative research between HICs and LMICs addressed relevant public health needs; (iii) the USA, China, the United Kingdom, and India have had the greatest influence on COVID-19 research in HIC–LMIC collaborations; (iv) authorship relationships between HICs and LMICs were more balanced, with research interests aligned with national expertise and global interests. The COVID-19 pandemic triggered new strategies, scientific approaches, and collaborations, but also highlighted the technological and scientific capacity gaps between HICs and LMICs. Advancing global health diplomacy is urgently needed to create a more equitable and scientifically collaborative world.

## Limitations

This study has limitations common to most bibliometric analyses. Not all research collaborations result in scientific publications, and not all co-authored publications are the result of true research collaborations. The Dimensions database, the source of publications used in this study, is considered relatively complete, but it may not include publications outside of the main editorial offices and may include preprints that are not published in scientific journals.

## Footnotes

1 https://console.cloud.google.com/marketplace/product/digitalscience-public/covid-19-dataset-dimensions.

2 https://console.cloud.google.com/bigquery?sq=571477072362:dd2eb2261ec5400aac075b1d534515f2.
